# Digital topics on cultural heritage investigated: how can data-driven and data-guided methods support to identify current topics and trends in digital heritage?

**DOI:** 10.1186/s43238-021-00045-7

**Published:** 2021-12-29

**Authors:** Sander Münster, Ronja Utescher, Selda Ulutas Aydogan

**Affiliations:** Digital Humanities, Friedrich Schiller University Jena, Jena, Germany

**Keywords:** Digital heritage, Research topics, Policies, Survey, Bibliometrics, NLP, Qualitative assessment

## Abstract

In research and policies, the identification of trends as well as emerging topics and topics in decline is an important source of information for both academic and innovation management. Since at present policy analysis mostly employs qualitative research methods, the following article presents and assesses different approaches – trend analysis based on questionnaires, quantitative bibliometric surveys, the use of computer-linguistic approaches and machine learning and qualitative investigations. Against this backdrop, this article examines digital applications in cultural heritage and, in particular, built heritage via various investigative frameworks to identify topics of relevance and trendlines, mainly for European Union (EU)-based research and policies. Furthermore, this article exemplifies and assesses the specific opportunities and limitations of the different methodical approaches against the backdrop of data-driven vs. data-guided analytical frameworks. As its major findings, our study shows that both research and policies related to digital applications for cultural heritage are mainly driven by the availability of new technologies. Since policies focus on meta-topics such as digitisation, openness or automation, the research descriptors are more granular. In general, data-driven approaches are promising for identifying topics and trendlines and even predicting the development of near future trends. Conversely, qualitative approaches are able to answer “why” questions with regard to whether topics are emerging due to disruptive innovations or due to new terminologies or whether topics are becoming obsolete because they are common knowledge, as is the case for the term “internet”.

## Introduction

Cultural heritage can be understood as traces and expressions from the past that are used in contemporary society (cf. UNESCO 1989). Cultural heritage can be regarded as the only legacy that cannot be inherited; instead, it must continuously be acquired (Kuhnke 2017). Since cultural heritage traditionally focuses on tangible objects, a broader understanding of adding intangible heritage and computer-based materials has become important over the last decade. This concept also includes digital cultural heritage materials, such as texts and images, which are created digitally or converted into digital form as well as digital resources of human knowledge or expression (e.g., cultural, educational, scientific) (UNESCO 2018).

The latter context also includes various digital technologies for studying cultural heritage. Various scholarly communities have formed around these topics in recent decades. In our previous research, we made some attempts to determine the boundaries of digital heritage studies as a scholarly field, e.g., with regard to the boundaries of adjacent scholarly fields such as digital humanities, digital archaeology or digital history studies or concerning related scholarly communities (Apollonio, F., S. Münster, H. Richards-Rissetto, F. Rinaudo, and R. Tamborrino: Exploring complementary overlap in digital humanities and digital heritage, in preparation; Münster et al. 2018, 2019). One main claim is that digital heritage consists of technologies to preserve, research and communicate cultural heritage (cf. Georgopoulos 2018).

Our overarching research question is as follows: **How can data-driven and data-guided methods support the identification of current topics and trends in digital heritage?**

The purpose of this paper is twofold. One interest is related to methodology. As a discipline, policy analysis still mainly uses qualitative analysis and quantitative modelling (cf. Browne et al. 2018) within case studies. Conversely, Big Data analytics that cover an entire sector rather than focusing on limited cases and text analytics that cover large collections of documents are still rarely used in policy studies, but with regard to adjacent domains (Agarwal and Dhar 2014), they may help gain more comprehensive and informed insights. In this research, we combine topic mining, trend analysis and pattern recognition technologies to track large-scale amounts of text, e.g., research publications and policy documents.

Regarding the complexity of the area of study, the methodical novelty of our approach lies in the combination of methods and scopes of analysis to enable cross-fertilisation and validation. This includes the qualification of patterns found via Big Data analysis, data-driven modelling and the interpretation of empirical findings. To investigate this topic, quantitative and qualitative empirical methods as well as linguistic analysis were used in four inductive corpus-based and deductive corpus-driven (Scharloth et al. 2013) stages of analysis:

- Survey among scholars: To examine the trends in and perspectives on digital heritage, we conducted a survey among 4500 scholars in digital heritage, receiving approximately 1000 responses.
- Scientometric analysis: To examine the core topics of and trends in digital heritage research, we analysed approximately 4500 articles from main conferences in the field of digital heritage.
- Natural Language Processing (NLP) and trend analysis: The use of large-scale text mining and text analysis is relatively new in innovation research and is primarily used in prototypical settings (Massey et al. 2013). A trend analysis of European Union (EU) Community Research and Development Information Service (CORDIS)^1^ data and research publications retrieved via the arXiv repository^2^ is used to examine the overlapping patterns and congruency of research policies and publications on digital heritage topics.
- Qualitative investigation of heritage policies: This stage involves a cultural heritage and digitisation-relevant policy framework, that is, policy analysis via a thematic inquiry.

The other interest is related to learning about trends and topics within the frameworks of research and policies. Historic environments are no longer considered merely obstacles to economic growth (Licciardi and Amirtahmasebi 2012). Cultural heritage is increasingly being recognised as contributing to economic added value, increased resilience, a reduction in ecological problems, the upgrading of neighbourhoods and increased property values (European Commission 2014; CHCfE Consortium 2015). Especially in the wake of the COVID-19 crisis, there is a huge demand for social and economic recovery and appropriate mechanisms to foster the resilience and sustainability of cultural heritage for innovation after the global COVID-19 outbreak comes to an end. While the changing role of cultural heritage is at present widely accepted, knowledge about concrete mechanisms, temporal and topic-related developments and measures concerning digitisation and cultural heritage is still lacking. Why is such knowledge important? A better understanding of the interplay between innovation and research in cultural heritage is an important prerequisite for understanding timing and success factors for a rewarding transformation. Therefore, this research may contribute to filling the information gap to better develop, implement and monitor policy actions for cultural heritage.

The study presented in this paper is exploratory, and it provides initial insights and findings within individual studies and in terms of an overarching methodology and cross-fertilisation across research frameworks. Sections 2 and 3 show global studies, while the studies described in Sections 4 and 5 are European studies.

## Survey of technological prospects

To analyse current demands from a community perspective, we conducted an online survey. Our specific interest was as follows: **What are the forecasts for technologies of relevance?**

### Related works

Various surveys have been conducted to investigate digital use and topics in the humanities and heritage studies. The Digital Research Infrastructure for the Arts and Humanities (DARIAH) Digital Methods and Practices Observatory (DiMPO) survey published in 2016 had 2100 participants and a specific focus on regional coverage and the use of digital methods (Digital Methods and Practices Observatory Working Group DARIAH-EU European Research Infrastructure Consortium 2016). The main findings were that the community in Europe is widely driven by German and French researchers. Similarly, the e-Science survey series with 860 participants covered the use of digital tools with regard to private and professional use. As one of its main findings, the use of digital tools for private use does not differ much between researchers from the humanities and researchers from other disciplines. Conversely, professional use is highly divergent between single humanities disciplines, but the tendency is lower than that in other disciplines (Albrecht 2013). In the context of digital heritage studies, various surveys on specific topics have been conducted. The Virtual Multimodal Museum (ViMM) survey with 700 participants queried digital challenges and protagonists (Münster et al. 2017). The INCEPTION project^3^ and the EUROPEANA 3D Task Force surveys (Fernie et al. 2020) were specifically on the use of 3D technologies. On an international basis, the authors studied the field via 3 panel surveys conducted since 2017 (Münster 2017).

### Methodology

Surveys are well-known instruments in the social sciences, and their principles, methods and practices have been well investigated (eg., Bhattacherjee 2012). The methodology of this survey was determined as follows:

- **Open-ended questions:** Due to our study’s interest in exploring the field, our survey used only open-ended questions to allow for diverse answers and to retrieve additional items (Reja et al. 2003).
- **Sampling:** The survey was sent to **~ 5000 individuals who were authors in the main conference in the field of digital cultural heritage as well as members of the International Centre for Archival Research (ICARUS), the International Centre for the Study of the Preservation and Restoration of Cultural Property (ICCROM) and Time Machine. The survey took place during** May and June 2019.
- **Survey participants: In total,** 968 participated, and 406 completed the survey. Since the questions were not dependent on each other, we also included only partially completed forms in the evaluation.
- **Data analysis:** Data clustering was performed by alternating inductive and deductive steps of qualitative content analysis (Mayring 2000).
- **Ethics:** All answers were provided anonymously. The acquired metadata contained location information only. These data were used to investigate the coverage of the survey.

### Findings

**Fig. 1:**
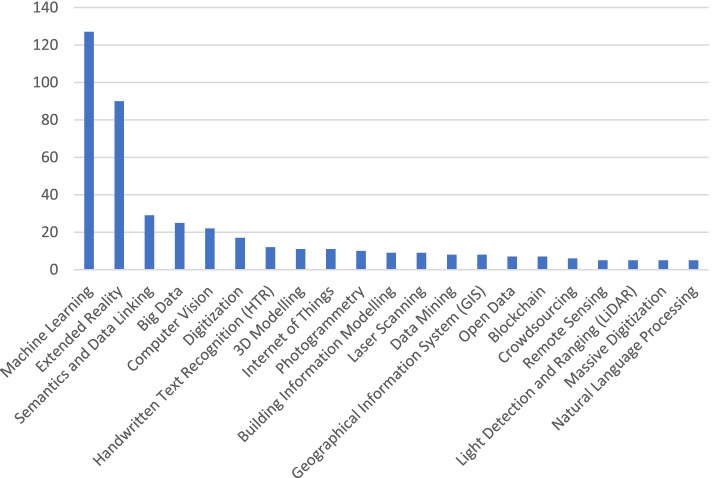
Question: “Which technologies would you suggest as most promising for your field of work?” Coding: 436 out of 995 answers—min. occurrence 5 per group (Source: the author)

Concerning the question on suggestions for promising technologies and demands, we received 995 answers by 377 individuals (Fig. 1).

Machine learning is the most frequently mentioned topic. It consists of various strands, such as artificial intelligence and deep learning, and specific technologies, such as generative adversarial networks (GANs). Extended reality, consisting of virtual reality, augmented reality, and mixed reality, is the second most frequently mentioned topic. Further technologies are mentioned with significantly lower numbers, such as semantics and data linkage, which consists of linked open data, the semantic web, and semantic processing. Big Data technologies rank fourth, and computer vision, including sub-categories such as image recognition, ranks fifth.

### Discussion

From the survey evolved a very clear view that machine learning and extended reality are currently assessed as the most promising technologies. This finding is in line with various European-scale endeavours, e.g., the strong emphasis on extended and mixed reality in the Horizon 2020 and Horizon Europe framework programmes (FPs) (e.g., DT-20,^4^ DT-12,^5^ DT-24^6^). In addition, various EU projects have assessed and developed mixed-reality approaches in cultural heritage, e.g., ViMM,^7^ 3D-Coform,^8^ and Inception^9^ (cf. Rigauts and Ioannides 2020).

It also seems interesting to examine the topics that were mentioned only occasionally. Data acquisition and digitisation technologies such as laser scanning, photogrammetry or light detection and ranging (LiDAR) are only occasionally noted as promising technologies, although—as discussed in the following paragraph—they were the most prominent topics at conferences during recent decades and even at conferences currently taking place (Münster 2019). In addition to specific technological strands, various overarching and non-technical concepts were named, among them open data, crowdsourcing and (massive) digitisation. This finding corresponds to the results of a qualitative survey of various expert meetings and conferences to identify current challenges in the field of digital heritage (Münster et al. 2015b), which noted the development of scientific transparency, standards and sustainable strategies for linking data as still unresolved major challenges.

Questionnaires are appropriate for easily examining a phenomenon without empirical data; therefore, as stated in this research, they allow not only retrospective analysis but also predictions. Despite these benefits, the reliability and validity of questionnaires are highly vulnerable to bias. These effects have been well investigated in many areas of research (e.g. Preisendörfer and Wolter 2014; Krosnick 1999; Bogner and Landrock 2015) and include phenomena related to both questionnaire design and methodology (e.g., with regard to acquiescence as “positive answering” (cf. eg. Rammstedt and Farmer 2013) or satisficing (cf. eg. Menold and Bogner 2015) as “overexposure of middle values”) or the respondent (e.g., social desirability (cf. eg. Krumpal 2013) and overexposure of short-term influences as current trends). As an alternative approach that is less vulnerable to these influences, the following stage is based on publications to assess the relevance of topics for current research.

## A bibliometric analysis of topics

A second stage was intended to identify relevant topics by studying publications. The underlying approach was taken from bibliometrics, which deals with the quantitative investigation of scientific structures and productivity based on publications (Egghe and Rousseau 1990).

### Related works

Regarding digital heritage, Scollar (1997) investigated the Conferences on Computer Application in Archaeologies (CAA) from 1971 to 1996 and the European Commission reports about projects completed under FP5-FP7 (European Commission 2011). Both studies found that researchers in the field of digital heritage are primarily located in Mediterranean countries and have backgrounds in various disciplines, including computing, the humanities, architecture and geo- and natural sciences. Concerning topics, Koutsabasis performed a literature-based survey about employed technologies and scenarios for interaction with cultural heritage (Koutsabasis 2017). According to his investigation, the most prominent scenarios are virtual museums and on-site presentations of cultural heritage. In addition to these quantitative studies, there have been various qualitative discussions (Ciolfi et al. 2017; Benardou et al. 2018). Regarding adjacent disciplines, a fundamental analysis of topics in the humanities was conducted by Leydesdorff et al. (2011) and most recently by Spinaci et al. (2021). For digital heritage studies, Spugnoli investigated the topics in Italian conference series (Sprugnoli et al. 2019). We studied these aspects by analysing 4500 publications stemming from six major conferences in digital heritage studies and dating from 1973 to 2015 (Münster 2019).

### Methodology

As examined within the publications described above, a key problem in all bibliometric research is the identification of relevant publications within a specific area. While standardised indexes such as the Arts & Humanities Citation Index (Thomson Reuter 2015) or the European Research Index in Humanities (ERIH) cover the whole range of disciplines, there are no indexes available specifically for the field of digital heritage. Thus, the first task consists of index construction for this field. Prior to creating a database, a survey with 988 participants in 2017 was conducted to identify and rank relevant journals and conferences in the field of digital heritage (Münster 2017).

In the analysis presented here, conference series were included. The sample was restricted to articles written in English and available electronically—these restrictions excluded specific issues (e.g., the 2008 CAA) available as printed proceedings only. The sample construction process has been discussed in previous articles (Münster 2019) and included the full set of articles meeting these requirements. The final sample presented in Table 1 included 4484 publications dating from 1973 to 2017.

**Table 1:** Sample (4484 articles)

Publication	Volumes (Sample B)	No. of Articles
3DArch Conf. (bi-annual conf.)^a^	2005–2017	389
CAA Conf. (annual conf.)^b^	1973–1992, 1994–2001, 2004–2009, 2011–2015	1637
VAST Conf. (annual conf.)^c^	2003–2006, 2008–2012	202
Digital Heritage (bi-annual conf.)^d^	2013, 2015	401
Euromed Conf. (bi-annual conf.)^e^	2006–2016	607
CIPA Conf. (bi-annual conf.)^f^	1999–2001, 2005–2017	1248

^a^Contributions published as special issues of ISPRS Archives: http://www.isprs.org/publications/archives.aspx, visited: 10.1.2018

^b^Proceedings published online: http://proceedings.caaconference.org/, visited: 10.1.2018

^c^Contributions published by Eurographics: https://diglib.eg.org/handle/10.2312/1003, visited: 10.1.2018

^d^Proceedings available via IEEE: http://ieeexplore.ieee.org/xpl/mostRecentIssue.jsp?punumber=6729393 (2013); http://ieeexplore.ieee.org/xpl/mostRecentIssue.jsp?punumber=7406203 (2015), visited: 10.1.2018

^e^Proceedings 2006-2008: https://www.euromed2018.eu/download_file/view/2323/241 Contributions from 2010 published in Springer LNCS Series

^f^Contributions published as special issues of ISPRS Archives and ISPRS Annals: http://www.isprs.org/publications/Default.aspx, visited: 10.1.2018

### Findings

The topics are tightly related to the technologies and disciplines of cultural heritage preservation and archaeology (Table 2). While nearly all publications mention remote sensing technologies, particular approaches such as laser scanning and photogrammetry are named in half of the articles. Presentation and visualisation issues such as virtual reality and user-centred design are mentioned by 87% of the articles, and aspects of rendering and visualisation are included in 82% of the articles. In contrast to survey results, data management and access aspects such as databases and interfaces are slightly less frequently mentioned, in total by 3/4 of the articles. Aspects of documentation and data linking, such as ontologies and semantics, are mentioned by only 21% of the articles. Nevertheless, there is a vivid community specialising in these latter topics. In addition, content was frequently mentioned. Regarding this aspect, archaeology and computing are frequently referred to. Additionally, conservation and cultural heritage aspects are mentioned in 85% of the articles. Built heritage and related technologies such as Building Information Modelling (BIM) are another important topic and are mentioned by 85% of the articles. Geo-based content and technologies such as geographical information systems (GISs) are also frequently mentioned by 70% of the articles.

**Table 2:** Topic map retrieved by factor analysis (*n* = 4484; the No. of publications included 15 out of 20 retrieved topics; keywords were not named manually but identified via exploratory factor analysis (cf. Kim and Mueller 1978))

Rank	Cases	% Cases	Name	Keywords
1	4189	93.42%	Remote Sensing	Remote Sensing; Photogrammetry; Spatial
2	3925	87.53%	Archaeology	Archaeology; Computing; CAA; Application
3	3903	87.04%	Presentation	Virtual; Interaction; Reality; Visitor; Exhibit; Environment; VR; Experience; Game; Museum; User
4	3865	86.20%	Modelling	Point; Coordinate; Distance; Camera; Measure; Position; Control; Calibration; Accuracy; Parameter; Error; Orientation
5	3850	85.86%	Cultural Heritage; Conservation	Heritage; Culture; Conservation; Preservation
6	3819	85.17%	Century; City	Century; City; Wall; Period; Roman; House
7	3803	84.81%	Architecture; Building	Architecture; Build; Element; BIM; Construction; Geometry; Structure
8	3695	82.40%	Visualisation	Texture; Resolution; Lighting; Render; Image; Colour; Surface
9	3485	77.72%	Data Management & Access	User; Database; Web; Interface; File; Access; Query
10	3161	70.50%	Geo Approaches	GIS; Geography; Landscape; Map
11	3035	67.69%	Automatic Algorithm	Algorithm; Match; Extract; Automation; Segment; Detect
12	2484	55.40%	Photo-based Approaches	Photography; Aerial; Photogrammetry; Photo
13	2081	46.41%	Laser Scanning	Laser; Scanner; Scanning; Cloud
14	1509	33.65%	Sensor & Devices	Sensor; Device
15	939	20.94%	Ontologies	Ontologies; Semantic; CRM; Metadata

Since a majority of articles include multiple topics, this characteristic of the articles may indicate a high level of cross-topic cooperation. Furthermore, topics such as remote sensing, presentation and data management are shown to be very important in both individual research topics and publication content. There are disciplinary links to computing, archaeology, geosciences and preservation. Similarly, architecture and landscapes are the most important contents, which matches findings of previous investigations (Münster et al. 2015a).

### Discussion

Regarding the findings of the bibliometric analysis, the community discourse is primarily about technologies and workflows, and new technologies are adopted early. For example, augmented reality, unmanned aerial vehicles (UAVs) and LiDAR were discussed at conferences and employed in projects shortly after their availability as ready-to-use technologies. Additionally, issues such as documentation, accuracy and semantics have been widely discussed by the community for many years. The topic mapping shows that temporal development has yet to be taken into consideration—this will be addressed by the research shown in the next stage.

## Data-driven trend analysis

The core intent underlying this work is to analyse academic trends and to move towards a model that can predict whether a topic will become (more) popular in the future. In general, there are two major aspects of such research: first, identifying topics of interest or finding appropriate signifiers of a topic and, second, analysing their prevalence in different academic contexts over time. This section focuses on the latter of the two.

### Related work

The use of large-scale text mining is relatively new in innovation research and is primarily used in prototypical settings (Massey et al. 2013). On a technical level, innovation research draws from text processing methods such as topic clustering (Aghabozorgi et al. 2015) and keyword extraction (Liu et al. 2010; Florescu and Caragea 2017). While many of these approaches are static, some consider topic development over time (Wang and McCallum 2006). However, much of the more recent work in this direction is in the social media domain (Salloum et al. 2017). Quantifying developments on platforms such as Twitter is superficially a similar task. However, the frequency of the data (relatively few and infrequent academic publications vs. a constant stream of tweets) and the nature of the vocabulary make trend mining a task with very different practical constraints.

### Data

The data used for the project are threefold: FP7 and FP8 project data, a subset of papers published on arXiv and author keywords from a list of digital object identifiers (DOIs). The FP7 and FP8 (Horizon 2020) datasets contain short descriptions and various metadata of approved EU-level research grants. These data are provided in two parts by CORDIS through the EU Open Data Portal.

The arXiv data cover the 1993–2018 period, whereas the CORDIS data cover the 2004–2020 period. However, it needs to be taken into account that the data for 2020 are incomplete and, consequently, much sparser than those of other years. Therefore, these data will be excluded from our analysis.

Both datasets contain the project/paper titles as well as short texts. Specifically, the project proposals contain a description of the project objective, whereas the arXiv dataset contains the papers’ abstracts.

Arxiv.org is an open-access archive for academic articles. For our model, we selected a random sample of the 1.8 million papers hosted in it.

Our model uses a sample of 810 unique author-defined keywords from the domain of digital cultural heritage. These keywords were scraped automatically from the Institute of Electrical and Electronics Engineers (IEEE), International Society for Photogrammetry and Remote Sensing (ISPRS), and Springer websites.

### Methodology

Keywords and topics can be extracted automatically; however, these methods typically cover only those keywords that achieve significant usage in the first place. Since this project is interested in differentiating between popular and unpopular topics, we decided to use author-defined keywords and to analyse their occurrence over time. As a first step towards analysing the behaviour of our now-retrieved keywords in both the arXiv and CORDIS data, we collected the frequencies of each keyword for each timestep. Due to the sparseness of the publication and project data, our analysis was performed using one-year intervals. Here, our primary metric is the normalised frequencies of terms. These normalised frequencies are expressed as a fraction of the absolute frequency over the number of documents at the given timestep.

We propose a prototype classification model that classifies keywords as either popular or unpopular. To train and evaluate such a classifier, it is necessary to provide a ground truth of what is and is not popular. We offer a simple heuristic: if the overall trend (as modelled by linear regression) is positive (> 0.05) overall, a keyword is considered popular. To avoid circularity, the models use only the arXiv data to predict the popularity in the CORDIS dataset. The model itself is a simple perceptron classifier that receives the normalised keyword frequency as input. Perceptrons are trained and evaluated on a set of features and the ground truth. In our case, the normalised (arXiv) keyword frequencies at each time step are the features, and our popularity heuristic provides the ground truth. Each feature is assigned a weight.^10^ During training, these weights are adjusted to reduce errors.

### Results

**Fig. 2:**
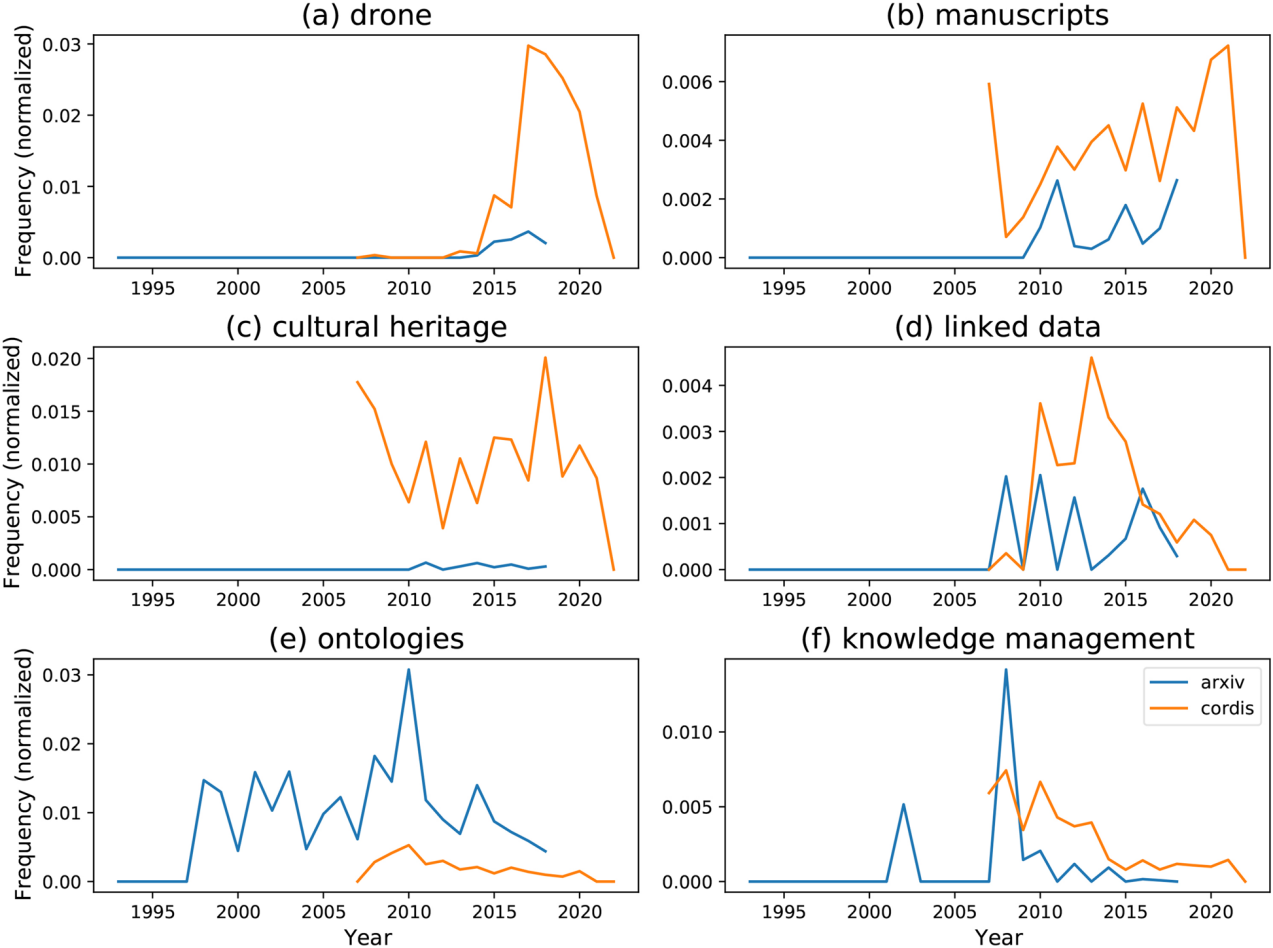
Examples of keyword frequency over time in the CORDIS and arXiv corpora (Source: the authors)

A preliminary quantitative analysis of the data revealed that the relationship between the popularity of a keyword in the EU research grant corpus and the arXiv corpus is, on average, not a straightforward correlation. There are a number of patterns. Some keywords are nearly exclusive to one of the corpora, e.g., digital heritage (Fig. 2c). Others show coinciding rises and falls in popularity, e.g., Fig. 2b. At the same time, these developments are not always of the same magnitude (e.g., Fig. 2a).

To train the classifiers, the popularity criteria are applied to each keyword. In our case, this application yields a 270/540 popular/unpopular split. We reserve a random 10% of both popular and unpopular keywords for testing. Table 3 shows the accuracy for all model types. Note that all of the models perform better than the majority baseline (0.666), with the perceptron performing the best (0.777).

**Table 3:** Classifier scores

	Perceptron	LogReg	SVM	Majority Baseline
Accuracy	**0.777**	0.703	0.728	0.666

### Discussion

The work presented in the section exemplifies two important challenges for quantitative trend research in general: (1) grounding what keywords are popular and (2) building a model that can integrate varying timespans of data.

For our measure of popular topics, it should first be reiterated that both heuristics introduced in the previous section make the assumption that a popular keyword is one that is used increasingly often in the CORDIS data. The underlying rationale is that being part of an approved EU project grant is itself a greater measure of success than “just” occurring in a publication.

As mentioned above, one major technical challenge in building a model is posed by the different timespans covered by the two corpora. It is possible to simply discard older publication data; doing so, however, would also entail a loss of information.

Fundamentally, our prototype models work by simply feeding the usage history of a keyword into a statistical model. The classifier scores signify that this sample of usage history can provide some information but is insufficient to completely explain its present usage. Consequently, future work in this direction could pursue broadening the amount of information that can be given to such models. After this data-driven view of EU policies, the next section will focus on investigating EU policies from a qualitative perspective.

## Qualitative investigation of heritage policies

This section examines the recent European policy framework concerning digitisation and cultural heritage. The most recent European-level policy documents, initiatives and programmes reflect a multi-dimensional approach to the digitisation of cultural heritage and the benefits to be acquired from it. This section focuses on research conceptualising cultural heritage associated with digitisation, innovation and the broader context of sustainability.

### Related works

Cultural heritage is mainly perceived as a factor in innovation, employment and development. In this respect, three main interrelated strands of conceptualisation in relation to the European policy framework were defined: 1) cultural heritage as a factor of sustainability; 2) cultural heritage for innovation and economic value; and 3) the institutional framework for cultural heritage and digitisation.^11^

1. Cultural heritage as a factor of sustainability is currently regarded as a common good and can be described as a process of change and constant flux (ICOMOS 2019). Cultural heritage is present in everyday society and is a resource linked to social capital, economic growth, and environmental sustainability (Bandarin and Van Oers 2014). With respect to this view, in the UN 2030 Agenda (Hosagrahar 2017), cultural heritage plays an important role in achieving most of the named global goals for sustainable development by enabling social cohesion and inclusion and be serving as a driver of equity and economic development.
2. The culture-driven production of meaning and skills is perceived as one of the main factors in a new logic of innovation and economic value generation. Currently, the spillover and cross-over effects of investments in cultural heritage are indisputable and have become a key asset at the top of any kind of value chain (Stanojev and Gustafsson 2019). Regarding its social effect, cultural heritage is understood as a “vector” for sustainable area development, where heritage determines the direction of spatial projects and developments (Janssen et al. 2017). The growth of creative content and modern digital technologies has given way to new conceptions, application fields, business models, initiatives, policies, projects, etc. The convergence of the cultural sector and modern technologies has triggered novel correlations among culture, the economy, society, technology and policy (Filip 2015).
3. The institutional framework of the European policy-making landscape has been subject to research from the perspective of legal settings in support of the digitisation of cultural heritage (Marinković et al. 2016), strategic approaches to cultural heritage as a domain of intervention by European cultural policy, and institutional rigidities in the digitisation of cultural heritage (Evens and Hauttekeete 2011), among others.

Policy analysis of the cultural heritage and digitisation domain mainly rests on and is fed by comparative policy and qualitative document analysis (Betzler and Fluturime 2019), presenting the normative EU framework for the digitisation of cultural heritage (Marinković et al. 2016), conducting an overview of European and national initiatives to monitor the state of digitisation (Bakker et al. 2011), surveying the state of digitisation across European cultural heritage institutions (Stroeker and Vogels 2014), and analysing the digitisation of cultural heritage and intellectual property through an interdisciplinary approach (Borissova 2018).

### Methodology

The most recent reference documents were retrieved through the culture and creativity, cultural heritage policies and initiatives website of the European Commission.^12^ Additionally, the European Commission’s Shaping Europe’s digital future, digital cultural heritage website^13^ was visited. The most recent policy and initiatives having contemporary effects on the digitisation of cultural heritage, research and innovation activities and framework conditions were analysed. Moreover, the Council of Europe, Culture and Cultural Heritage website^14^ was analysed to obtain contemporary policy references and initiatives. For this exercise, 15 policy reference documents having contemporary effects were analysed. Qualitative content analysis was applied to understand and articulate the content and main reference points of the policy documents with regard to digitisation, research and innovation.

In an attempt to conduct a qualitative investigation of current digitisation and cultural heritage policies, the conceptual basis of the recent European policy framework is discussed. This discussion is coupled with an examination of state-of-the-art research on policy analysis.

### Findings

The current European policy framework of digitisation and cultural heritage is based on a set of strategies, initiatives and programmes. They grasp cultural heritage mainly in relation to education, tourism, sustainability, development, competitiveness and job creation. Additionally, the most recent policy objectives reflect a multi-dimensional approach in support of cultural heritage-led innovations.

The policy framework in support of the digitisation of cultural heritage and the innovations driven by it gained a new momentum in search of strategies to combat the negative effects of the COVID-19 pandemic. This new momentum is based on support for the further digitisation of cultural heritage institutions and relevant sectors for their recovery and response to adverse effects of the crisis caused by the pandemic. In 2020, the European Commission launched calls with the aim of helping the digital transformation of museums and cultural institutions and helping invigorate the interregional ecosystems for digital and sustainable tourism as part of the response to and recovery from the COVID-19 crisis.^15^ Additionally, the new EU Research and Innovation Framework Programme Horizon Europe has an individual domain, Cluster 2 “Culture, Creativity and Inclusive Society”, which provides more space for cultural heritage-, digitisation- and innovation-related actions (European Commision 2019a).

Moreover, the European Commission launched a public consultation to collect stakeholders’ views on digitisation in cultural heritage and on the Commission’s recommendation on the digitisation and online accessibility of cultural material and digital preservation (2011/711/EU).^16^ The consultation also aimed to understand the impact of the COVID-19 crisis on the sector and how stakeholders perceive the role of digitisation of cultural heritage under these circumstances.^17^This could be seen as an attempt to restructure the European policy framework concerning the digitisation of cultural heritage in the context of the challenges posed by the pandemic.

Most recent policy documents, programmes and initiatives that have contemporary influence on the European policy framework for the digitisation of cultural heritage mostly aim to enable an aligned policy landscape and favourable framework conditions and ecosystems. These include *The European* *Cultural Heritage Strategy for* *the* *21*^*st*^ *Century* (Council of Europe 2017), *A New European Agenda for Culture* (European Commision 2018), *The Work Plan for Culture 2019–2022* (Council of the European Union 2018), The European Year of Cultural Heritage 2018, which boosts community engagement and the role of cultural heritage across a wide body of stakeholders in Europe and enables evidence-based policy-making (European Commision 2019b), and The Declaration of Cooperation on advancing digitisation of cultural heritage (launched in 2019, Digital Day).^18^

Moreover, The Recovery Plan for Europe (European Commission 2020), aims to address the adverse effects of the pandemic in economic and social terms and to determine way out of it, rests on the EU’s long-term budget and NextGenerationEU as a temporary tool to foster recovery. It mentionsl of “...a greener, digital and more resilient economy and society...” (European Commission 2020). The Plan makes mention of digital transitions, including the culture and tourism sectors. Resources will be allocated for digital transitions and capabilities to address the impact of the crisis for resilience and recovery.

The recent European policy framework on digitisation and cultural heritage is mainly based on four axes. The first axis concerns boosting the innovations driven by cultural heritage and the social and economic benefits proposed by it. The second has to do with raising and sustaining institutional and legal frameworks in support of the digitisation of cultural heritage, and the third is about helping to reverse the negative effects of COVID-19 on cultural heritage-relevant sectors through digitisation. A further cross-cutting strand could be identified around the topic of sustainability.

Sustainability is at the crossroads of innovation, development, and resilience aspects, which appear to be at the forefront of the EU policy context. This process could be assessed as having been triggered by recovery efforts against the COVID-19 crisis.

Within this policy framework, the ViMM project’s Manifesto,^19^ Roadmap and Action Plan^20^ of 2019 provide a vision and a set of measures for a future strategy and practicality for virtual museums and digital cultural heritage in a five-year span. There is an emphasis on assisting the European Commission and other public bodies in the decision-making process, including the funding context. This output is an example of EU-funded projects that, in a bottom-up manner, feed into decision and funding strategy processes at the EU scale.

## Conclusion

What can we learn from the multi-method analysis of the trends in and topics of digital heritage? Some preliminary findings are:

As stated in the previous analysis, research projects highly depend on technological trends and the advent of new technologies (Münster and Ioannides 2015). This finding contrasts with the long-term relevance of overarching concepts, e.g., “cultural heritage”, “geo-based approaches” and “architecture”, as scholarly domains or “open data” as conceptual approaches. Regarding the comparison of research and policy documents in Section 3, interestingly, these meta-concepts are frequently mentioned in policy papers but rarely used as descriptors of research work. Concerning the results of Section 2, research papers use more specific and particular descriptors. A resultant assumption is that policy papers more frequently use high-level and non-technical concepts than research papers.

Another question concerns the technologies of rapidly changing “disruptive” prominence, such as machine learning or extended reality. Such technologies are assessed as most relevant in the survey in Section 1 but were not traceable in either the bibliometric study in Section 2 or—prior to 2015—the policy papers investigated in Section 3. Since the topic of machine learning gained breakthrough momentum through the practical application of convolutional neural networks for computer vision in 2012 (Krizhevsky et al. 2012), extended reality has appeared as a terminological replacement of the previously used virtual and augmented reality. Both examples are significant due to the limitations of current automated evaluation strategies. Topic mining may reveal the proximity of concepts such as virtual, augmented, mixed and extended reality and their link to computer visualisation. However, an answer to the “why” questions regarding the link between those terms is lacking—did one term replace, extend, or diminish another and does it represent a completely new concept or is just a new iteration of an old concept? This kind of answer can currently be provided only by qualitative empirical studies, as shown in Section 4.

A third example is related to the influence of policies on research topics. The cross-national collaboration in research intended by the European Commission and highlighted in Section 4 has already led to cooperation patterns in research publications that differ from those of other continents: The fertilizers are no longer proximity or the same language of the co-authors but their location within the EU. This aspect was studied and quantified via a bibliometric analysis and described in a previous publication (Münster 2019). It will be interesting to see whether the current policy interventions sketched in Section 4—such as the strong link to impact and valorisation in current programmes such as EU Horizon Europe or the COVID-19 Recovery Funds—will shape research topics and cooperation patterns in a way similar to the cross-national cooperation required for projects in many EU funding programmes. This topic may be studied via a bibliometric analysis (Section 2) and with regard to temporal patterns via NLP (as shown in Section 3).

What do these findings mean with regard to technologies? The findings show the potential gain of a mixed-methods approach, where the results obtained via one method can enhance the results obtained via another approach and vice versa. Since this paper currently presents a couple of first findings, our interest and proposed next steps are to assess the extent to which and under which conditions the meaning and quality of findings can be amended by a mixed-methods approach. Finally, we are interested in assessing the boundaries of data-driven approaches with regard to the currently missing answer to “why”.

Within this framework, the question of the interface between policy and research proposes methodological enrichment and challenges. For a data-driven policy analysis that shows the community perspective, scientific and project topic trends complement the qualitative content analysis of the policy content and messages. The challenge is to derive insightful conclusions from the data and findings obtained via quantitative methods based on the timewise correlation of research and project topic trends to better identify the pathways and direction of influence. Moreover, the topic popularity aspect can be coupled with a more granular analysis of emerging topics (low in popularity but with a high potential of growth) and tested against community needs for a possible translation into the policy space. Doing so would also require an analysis of interdisciplinary networks and nodes emerging via publication and project collaborations.

The findings of this article show that the digital heritage research community sees advanced technologies that are promising for this field. The bibliometric analysis shows that technologies such as remote sensing, laser scanning and photogrammetry are mostly referred to in publications. Interdisciplinary linkages are observed across the humanities, computing, archaeology, the natural sciences, among others. Coupling these findings with FP7 and Horizon 2020 data is a challenge, as the CORDIS data provide information on abstracts and project titles that may not provide an opportunity to grasp the full picture of the technological references of the projects in question.

The current move towards digitisation and the use of advanced technologies, which is observed in the policy documents and programmes in relation to digitisation and cultural heritage, seems to couple with the prospects of the research community. Additionally, research outputs show that new technologies are adopted early. Interdisciplinary research collaborations exist. On this basis, the question of sustainability in the context of the digitisation of cultural heritage as a cross-cutting policy axis, together with the other four defined in this paper, could be revisited. The research and innovation nodes, research outputs and innovations driven by digitisation and cultural heritage, innovation diffusion and adoption patterns would be among the sustainability-relevant contemporary issues in the European policy framework.

A data-driven analysis of the dependencies between policies and research is an important prerequisite for supporting evidence-based policy-making with tools and insights for policy-makers to better manage innovations in digital cultural heritage for enhanced sustainability. Especially in the current situation of the COVID-19 pandemic, such an analysis may support a quick and efficient policy response, which is important for economic and societal recovery.

## Data Availability

Data publication is foreseen for non-copyright protected and anonymised material.

## Abbreviations

BIM: Building information modelling
FP: Framework programme
GAN: Generative adversarial network
GIS: Geographical information system
NLP: Natural language processing

## Footnotes

1
https://cordis.europa.eu/

2
https://arxiv.org/

3
https://inception-project.eu

4 DT-TRANSFORMATIONS-20-2020-European Competence Centre for the preservation and conservation of Monuments and Site: https://ec.europa.eu/info/funding-tenders/opportunities/portal/screen/opportunities/topic-details/dt-transformations-20-2020

5 DT-TRANSFORMATIONS-12-2018-2020-Curation of digital assets and advanced digitization: https://ec.europa.eu/info/funding-tenders/opportunities/portal/screen/opportunities/topic-details/dt-transformations-12-2018-2020

6 DT-TRANSFORMATIONS-24-2020: European Museum Collaboration and Innovation Space: https://ec.europa.eu/info/funding-tenders/opportunities/portal/screen/opportunities/topic-details/dt-transformations-24-2020

7 ViMM Project Website: https://www.vi-mm.eu/

8 3D-Coform Project Website: https://web.archive.org/web/20180514102115/https://www.3d-coform.eu/

9 Inception Project Website: https://www.inception-project.eu/en

10 Weights reflect the feature’s relevance to the classification. The classification decision is made based on whether the sum of the weighted inputs is > 0.

11 Please also see (Loebbecke and Thaller 2005) for a literature review and classification of the literature in relation to the digitisation of cultural heritage.

12 European Commission, Culture and Creativity, Cultural heritage: https://ec.europa.eu/culture/cultural-heritage

13 European Commission, Shaping Europe's Digital Future, Digital Cultural Heritage: https://digital-strategy.ec.europa.eu/en/policies/cultural-heritage

14 Council of Europe, Culture and Cultural heritage, Cultural heritage at the Council of Europe: https://www.coe.int/en/web/culture-and-heritage/cultural-heritage

15 One of the mentioned calls launched under Horizon 2020 is DT-TRANSFORMATIONS-24-2020 European Museum Collaboration and Innovation Space, reachable via https://ec.europa.eu/info/funding-tenders/opportunities/portal/screen/opportunities/topic-details/dt-transformations-24-2020. The other is the Pilot Action on Interregional Innovation Call for Expression of Interest for COVID-19 Response and Recovery Partnerships. It involves sustainable and digital tourism as one of its thematic focuses and is reachable via Publication of Calls for expressions of interest - DG REGIO - Regional Policy - European Commission to be supported under the European Regional Development Fund (ERDF) available at: https://ec.europa.eu/regional_policy/en/newsroom/funding-opportunities/calls-for-expressions-of-interest/

16 The public consultation on digitisation and the online access of cultural material and digital preservation is reachable via https://ec.europa.eu/info/law/better-regulation/have-your-say/initiatives/11837-Evaluation-of-the-Recommendation-on-digitisation-and-online-accessibility-of-cultural-material-and-digital-preservation/public-consultation

17 The Factual Summary Report on the open public consultation on digital for Cultural Heritage is reachable via https://ec.europa.eu/info/law/better-regulation/have-your-say/initiatives/11837-Evaluation-of-the-Recommendation-on-digitisation-and-online-accessibility-of-cultural-material-and-digital-preservation/public-consultation

18 European Commission, Shaping Europe's digital future, DIGIBYTE | Publication 09 April 2019, EU Member States sign up to cooperate on digitising cultural heritage: https://digital-strategy.ec.europa.eu/en/news/eu-member-states-sign-cooperate-digitising-cultural-heritage

19 The ViMM Manifesto for Digital Cultural Heritage available at: https://www.vi-mm.eu/vimm-results/

20 ViMM Project, 13 March 2019, Deliverable 7.3: Roadmap and Action-Plan available at: https://www.vi-mm.eu/vimm-results/
